# Supplementary material for: Fast in-line failure analysis of sub-micron-sized cracks in 3D interconnect technologies utilizing acoustic interferometry
Source: Commun Eng. 2024 Jul 19;3:100. doi: 10.1038/s44172-024-00247-8 (PMC11271500; doi:10.1038/s44172-024-00247-8)
Supplement: Supplementary file 2 — Supplementary Information [file 44172_2024_247_MOESM2_ESM.pdf]

Supplementary Information

Fast In-Line Failure Analysis of Sub-micron-sized Cracks in 3D Interconnect Technologies Utilizing Acoustic Interferometry

Priya Paulachan[1], Rene Hammer[1], Joerg Siegert[2], Ingo Wiesler[3], Roland Brunner[1*]

[1] Materials Center Leoben Forschung GmbH, Leoben, Austria

[2] ams-OSRAM AG, Premstaetten, Austria

[3] PVA TePla Analytical Systems GmbH, Westhausen, Germany

*Corresponding author E-mail: roland.brunner@mcl.at

Supplementary Note 1: Calculation of Rayleigh wave velocity from fringe spacing

When the SAM interferometry setup is deliberately set out of focus ($Z > Z_0$), distinct fringes emerge, spaced apart by half of the Rayleigh wavelength $\lambda_R/2$ around the TSV. These fringes allow for the calculation of the Rayleigh wave velocity, which can be determined through the application of Equation (1) and Equation (2)[1,2].

$$Rayleigh\ Wavelength, \lambda_R \ = 2 \times Fringe\ Spacing(\Delta x) \quad (1)$$

$$Rayleigh\ velocity, V_R \ = f \times \lambda_R \quad (2)$$

As we increase the defocus distances (Z_1, Z_2, ..., Z_n), as illustrated in **Supplementary Figure 1a**, a noticeable increase in the number of oscillations within the intensity becomes apparent. When we examine a cross-sectional slice (xx') taken at 0° in the SAM C-scan images and generate an intensity plot along this cross-section, as shown in **Supplementary Figure 1b**, we gain the ability to compute Rayleigh wave velocities based on the spacing between fringes at each defocus position. It is advantageous to consider the fringes nearest to the circumference of the TSV, as they provide higher contrast thus facilitate accurate Rayleigh wave velocity calculation[3].

At focus position, almost no oscillations are observed, indicating nearly almost zero Rayleigh wave velocity. Conversely, for Z_1 to Z_{12} the Rayleigh wave velocity is calculated using Equation 1 and Equation 2. The procedure was repeated for four additional TSVs, and the Rayleigh wave velocities based on fringe spacing at each defocus positions was calculated. The mean values

of these calculated Rayleigh wave velocities across all defocus positions are summarized in Table S1.

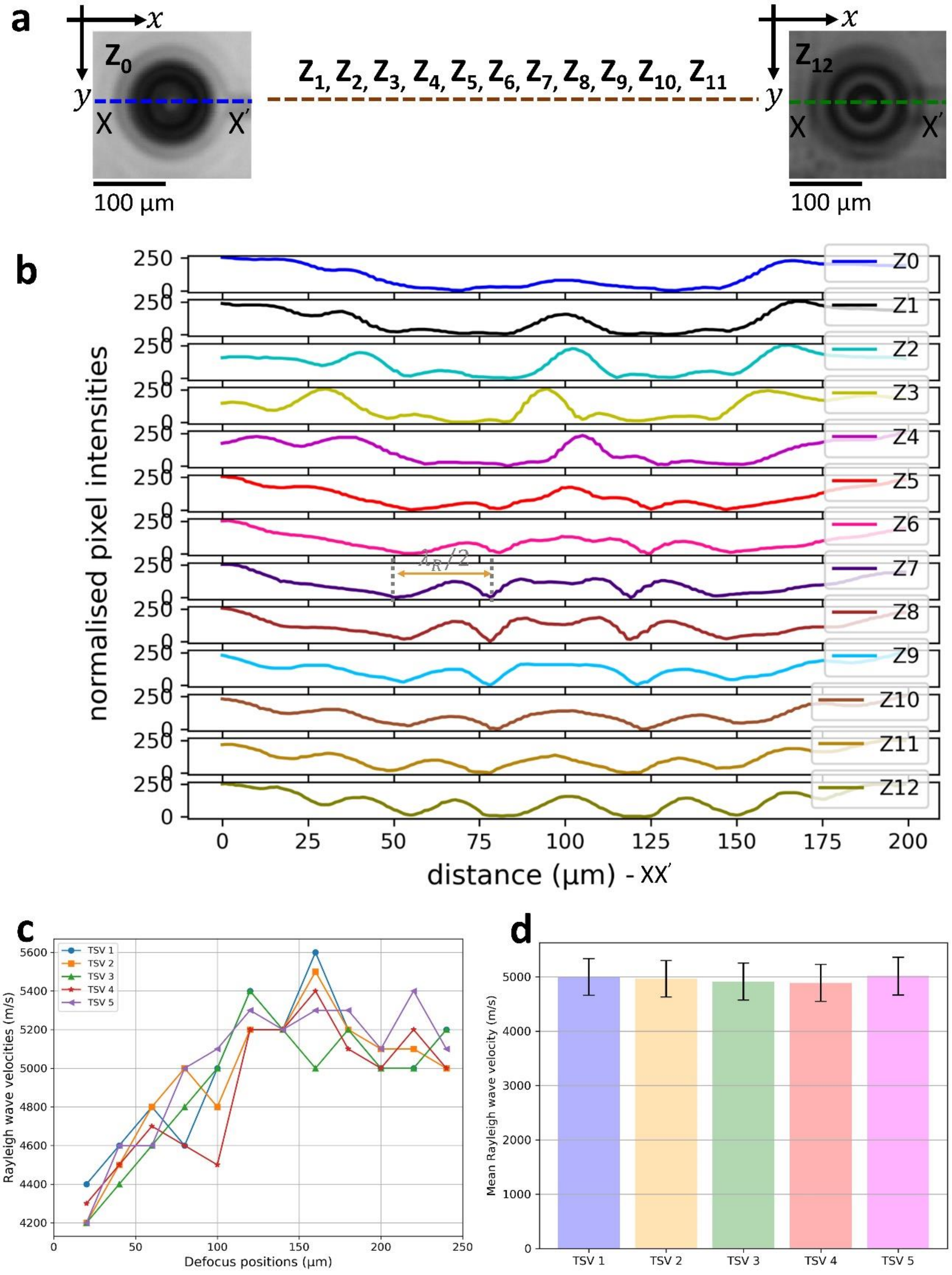

Supplementary Figure 1. **Extraction of Rayleigh wave velocity from SAM C-scan interferometric fringes of five TSVs at different defocus positions. (a)** The SAM C-scan images obtained at positions from Z_0, Z_1, Z_2, …, Z_{12} . A consistent cross-sectional reference line (XX') at the same location is considered, facilitating the extraction of Rayleigh wave velocity from the width of TSV fringes. **(b)** The normalized pixel intensity profiles along the cross-sectional line

(XX') for 13 Z –positions (Z_0 to Z_{12}). **(c)** Plot illustrating the calculated Rayleigh wave velocities from fringe spacing at various defocus positions for five TSVs exhibiting homogeneous fringes. **(d)** Mean Rayleigh wave velocity for each TSVs over all the defocus positions.

Unlike other defocus positions, where the calculated Rayleigh wave velocities varies, there is a consistent Rayleigh wave velocity of 5200 m/s at defocus position Z_7 (-140 µm) for all the five TSVs, see **Supplementary Figure 1c**. Therefore, we have chosen Z_7 as particular imaging position for further analysis, given the favorable tradeoff between contrasts at different defocus positions.

Supplementary Table 1. Mean Rayleigh wave velocities from the SAM C-scan interference fringes of three TSVs across multiple defocus positions.

**TSV**	**Mean V_R (m/s)**
TSV 1	5000
TSV 2	4967
TSV 3	4916
TSV 4	4891
TSV 5	5016

Based on these analyses of five TSVs at 12 defocus positions, the calculated mean Rayleigh wave velocity approximates 5000 m/s, closely aligning with the theoretical Rayleigh wave velocity in silicon. This quantitatively demonstrates the excitation of SAW at positions below the focus position.

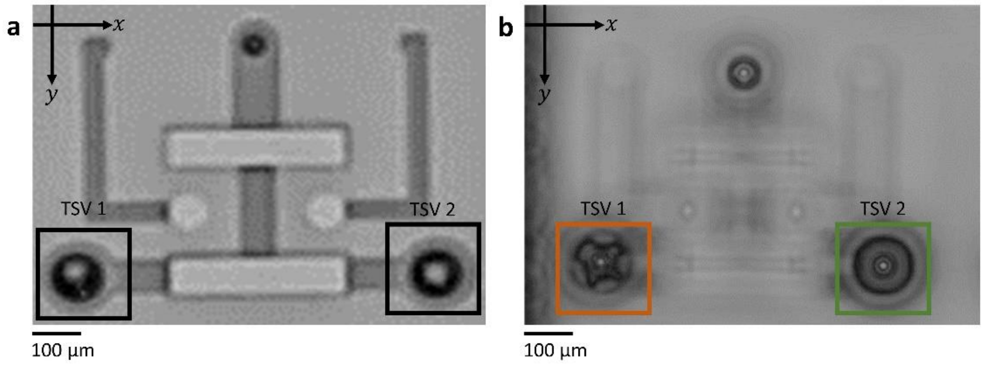

Supplementary Figure 2. Comparison of SAM C-scan images of TSVs. The C-scan images with 100 MHz of the identical TSVs (TSV 1 and TSV 2) are obtained using **(a)** conventional SAM setup and **(b)** SAM interferometric setup. The TSVs showing interference patterns with and without inhomogeneities are represented in orange and green color, respectively. No characteristic interference patterns are visible for the conventional C-scan SAM image.

Supplementary Figure 2 a and **b** shows the comparative analysis of SAM C-scan images representing the same TSVs, acquired through conventional SAM and SAM interferometry setups, respectively with a frequency of 100 MHz. In the conventional SAM setup (**Supplementary Figure 2a**), the acoustic lens is focused on the sample surface. However,

unlike the SAM interferometry setup (**Supplementary Figure 2b**), the SAM C-scan images from the conventional setup lack information about the TSVs' quality. Notably, in **Supplementary Figure 2b**, TSV1 and TSV2 exhibit interference fringes, providing valuable insights into the quality assessment of the TSVs. This observation has been corroborated through validation with SEM microscopy, where the blossom-shaped pattern corresponds to defective TSVs, while the concentric pattern signifies non-defective TSVs.

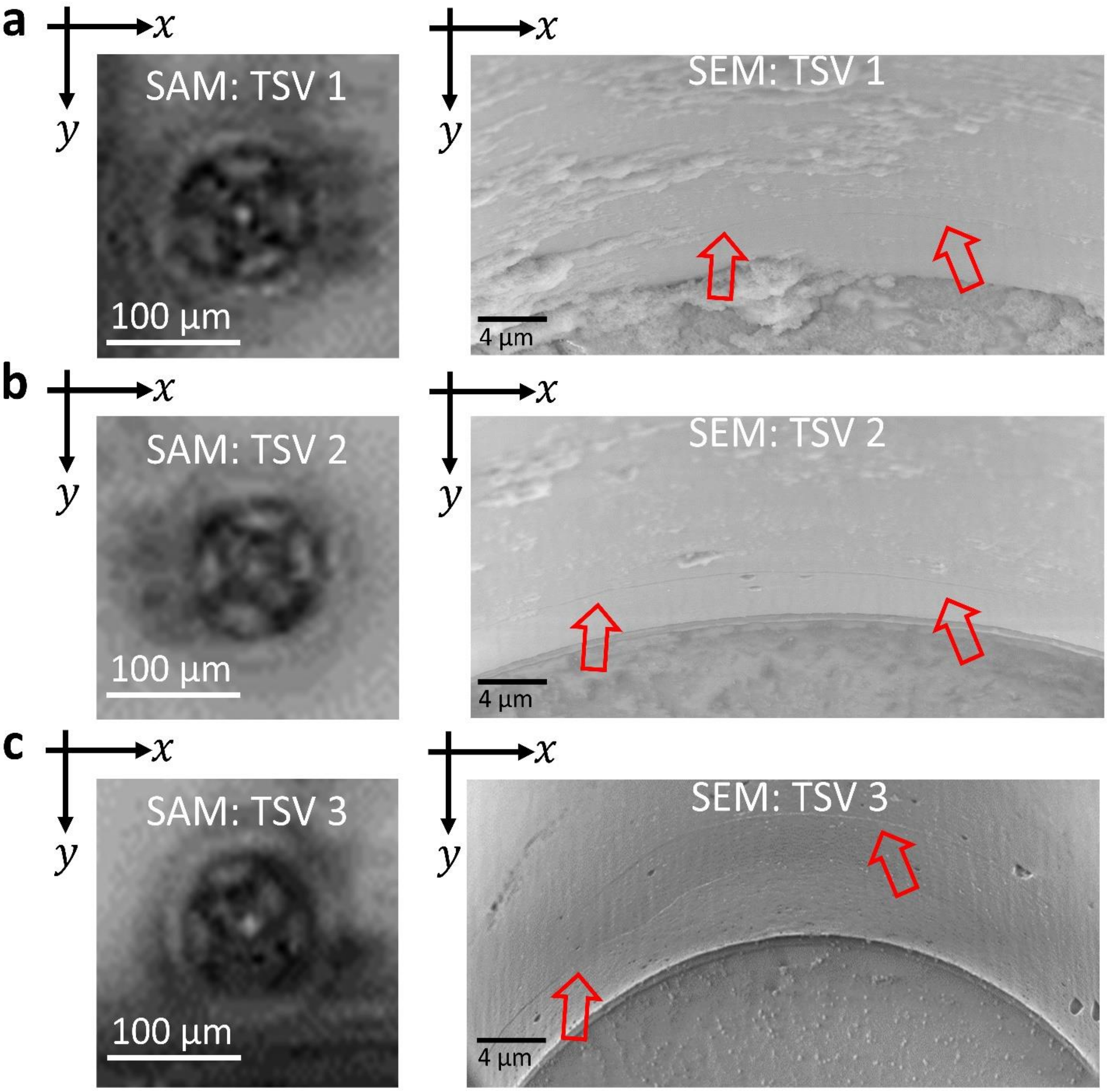

Supplementary Figure 3. **Detection of circumferential cracks within TSVs.** The SAM C-scan image of **(a)** TSV 1, **(b)** TSV 2 and **(c)** TSV 3 with blossom shaped interference pattern corresponding to sidewall crack in the SEM images.

Supplementary Note 2: Basic equations for 3D Elastodynamic Finite Integration Technique (EFIT) based simulations in differential form.

For simulating the elastic wave propagation using EFIT, we consider the entire material composed of several grids, as $'n'$, $'n+1'$, **see Supplementary Figure 4**. $'n'$ and $'n+1'$ represent the current and adjacent cell to the right respectively. The unit cell for 3D-EFIT simulation with velocity and stress components is defined at more than one location in the grid (staggered scheme), see **Supplementary Figure S4**. The wave field's velocity and stress components have been then positioned to improve the grid resolution and reduce numerical dispersion errors[4,5].

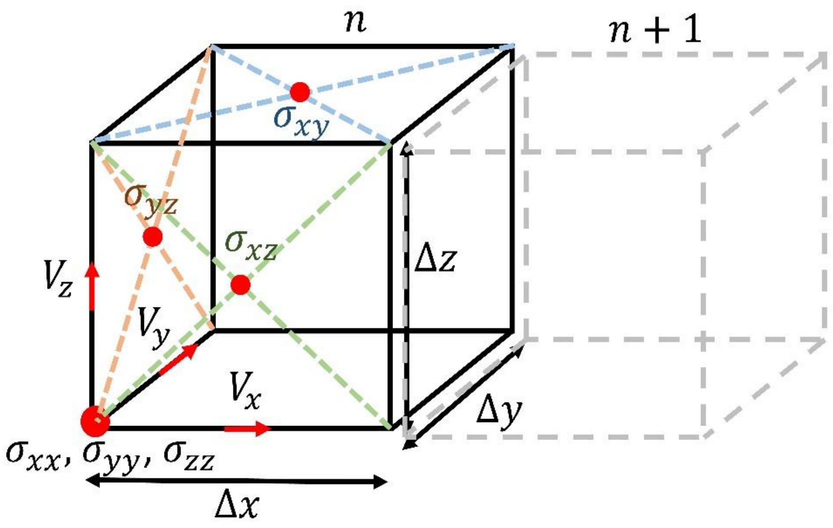

Supplementary Figure 4. Staggered grid of 3D Elastodynamic Finite Integration Technique (EFIT) Simulation.

Cauchy's equation for motion and Hooke's law describes the elastic wave propagation in an isotropic media[4,6],

$$\rho \frac{\partial v}{\partial t} = \nabla \cdot \sigma + f \quad (3)$$

$$\sigma = \lambda \epsilon \delta + 2\mu\epsilon \quad (4)$$

where ρ is the mass density, $v = (v_x, v_y, v_z)$ is the particle velocity, $\sigma = \{\sigma_{i,j}\}_{i,j=x,y,z}$ is the stress tensor, $f = (f_x, f_y, f_z)$ are the body forces, λ and μ are the Lamés parameter, and ϵ is the strain tensor. The Lamés parameters related to longitudinal (C_L) and transverse (C_T) speed of sound by the following equations.

$$C_L = \sqrt{(\lambda + 2\mu)/\rho} \quad (5)$$

$$C_T = \sqrt{\mu/\rho} \tag{6}$$

By arranging the velocity components on the edges of the unit cell together with the diagonal elements of the stress tensor on the corners and off-diagonal elements on the faces of unit cell, Equations (3) and Equations (4) can be discretized[4,7,8] as follows.

$$\dot{v}_x(t) = \frac{1}{\rho}\left[\frac{\sigma_{xx}^{(n+\hat{x})}(t)-\sigma_{xx}^{(n)}(t)}{\Delta x} + \frac{\sigma_{xy}^{(n)}(t)-\sigma_{xy}^{(n-\hat{y})}(t)}{\Delta y} + \frac{\sigma_{xz}^{(n)}(t)-\sigma_{xz}^{(n-\hat{z})}(t)}{\Delta z}\right] + f_x\,(t) \tag{7}$$

$$\dot{v}_y(t) = \frac{1}{\rho}\left[\frac{\sigma_{xy}^{(n)}(t)-\sigma_{xy}^{(n-\hat{x})}(t)}{\Delta x} + \frac{\sigma_{yy}^{(n+\hat{y})}(t)-\sigma_{yy}^{(n)}(t)}{\Delta y} + \frac{\sigma_{yz}^{(n)}(t)-\sigma_{yz}^{(n-\hat{z})}(t)}{\Delta z}\right] + f_y\,(t) \tag{8}$$

$$\dot{v}_z(t) = \frac{1}{\rho}\left[\frac{\sigma_{xz}^{(n)}(t)-\sigma_{xz}^{(n-\hat{x})}(t)}{\Delta x} + \frac{\sigma_{yz}^{(n)}(t)-\sigma_{yz}^{(n-\hat{y})}(t)}{\Delta y} + \frac{\sigma_{zz}^{(n+\hat{z})}(t)-\sigma_{zz}^{(n)}(t)}{\Delta z}\right] + f_z\,(t) \tag{9}$$

$$\dot{\sigma}_{xx}^{(n)}\,(t) = (\lambda + 2\mu)\,\frac{v_x^{(n)}(t)-v_x^{(n-\hat{x})}(t)}{\Delta x} + \lambda\left(\frac{v_y^{(n)}(t)-v_y^{(n-\hat{y})}(t)}{\Delta y} + \frac{v_z^{(n)}(t)-v_z^{(n-\hat{z})}(t)}{\Delta z}\right) \tag{10}$$

$$\dot{\sigma}_{yy}^{(n)}\,(t) = (\lambda + 2\mu)\,\frac{v_y^{(n)}(t)-v_y^{(n-\hat{y})}(t)}{\Delta y} + \lambda\left(\frac{v_x^{(n)}(t)-v_x^{(n-\hat{x})}(t)}{\Delta x} + \frac{v_z^{(n)}(t)-v_z^{(n-\hat{z})}(t)}{\Delta z}\right) \tag{11}$$

$$\dot{\sigma}_{zz}^{(n)}\,(t) = (\lambda + 2\mu)\,\frac{v_z^{(n)}(t)-v_z^{(n-\hat{z})}(t)}{\Delta z} + \lambda\left(\frac{v_x^{(n)}(t)-v_x^{(n-\hat{x})}(t)}{\Delta x} + \frac{v_y^{(n)}(t)-v_y^{(n-\hat{y})}(t)}{\Delta y}\right) \tag{12}$$

$$\dot{\sigma}_{xy}^{(n)}\,(t) = \mu\left(\frac{v_x^{(n+\hat{y})}-v_x^{(n)}}{\Delta y} + \frac{v_y^{(n+\hat{x})}-v_y^{(n)}}{\Delta x}\right) \tag{13}$$

$$\dot{\sigma}_{xz}^{(n)}\,(t) = \mu\left(\frac{v_x^{(n+\hat{z})}-v_x^{(n)}}{\Delta z} + \frac{v_z^{(n+\hat{x})}-v_z^{(n)}}{\Delta x}\right) \tag{14}$$

$$\dot{\sigma}_{yz}^{(n)}\,(t) = \mu\left(\frac{v_y^{(n+\hat{z})}-v_y^{(n)}}{\Delta z} + \frac{v_z^{(n+\hat{y})}-v_z^{(n)}}{\Delta y}\right) \tag{15}$$

The variables $\hat{x}$,$\hat{y}$, $\hat{z}$ are the unit steps in the x, y and z directions and n denotes the current cell. Using the central difference operator, the time derivatives in Equation (7) to Equation (15) are approximated yielding a leap-frog scheme[4]. In our case, we have an inhomogeneous medium with different material parameters. Therefore, we have to discretize the material parameters as well[9].

Supplementary Note 3: Relation between Time of Flight (ToF) and depth.

The distance between the transducer and silicon surface $'d'$ can be determined from the A-scan signal using the following equation,

$$d = \frac{\mathrm{ToF} \times \mathrm{c}}{2} \tag{16}$$

Where $'\mathrm{ToF}'$ is the time of flight and $'\mathrm{c}'$ is the speed of sound in water.

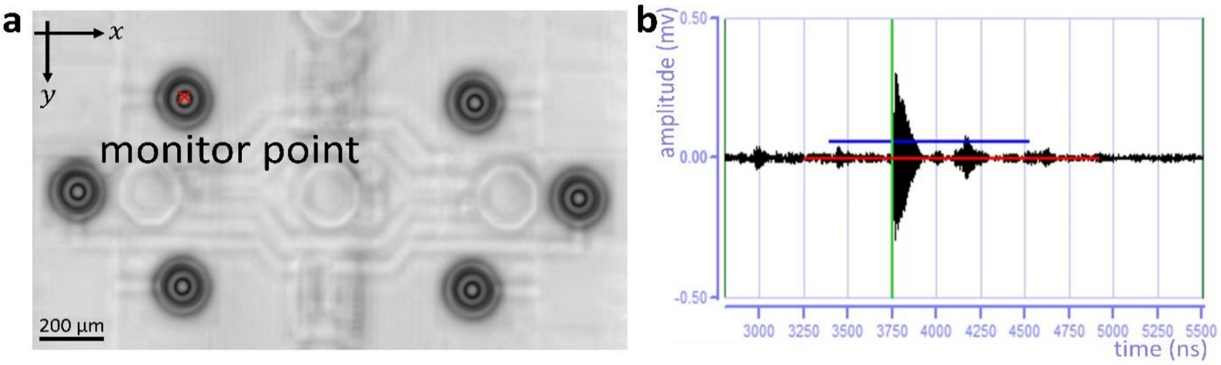

Supplementary Figure 5. To determine the transducer position from SAM C-scan image and A-scan signal (a) SAM interferometric C-scan image with six TSVs. A monitor point is marked to extract the A-scan signal from that point. **(b)** The A-scan signal corresponds to the monitor point with a gate (red color) selected to examine the specific time in the echo.

From the A-scan signal, we can determine the time taken by the acoustic wave travelling with a speed of approximately 1500 m/s to hit the substrate (silicon) and to return to the transducer as 3750 ns (ToF). This time of flight information extracted from the A-scan signal can be leveraged to calculate the height of the transducer using Equation (16).

When the ToF as well as speed of sound in water are plugged into the Equation (16), the position of the transducer is calculated as 2812 µm from the substrate.

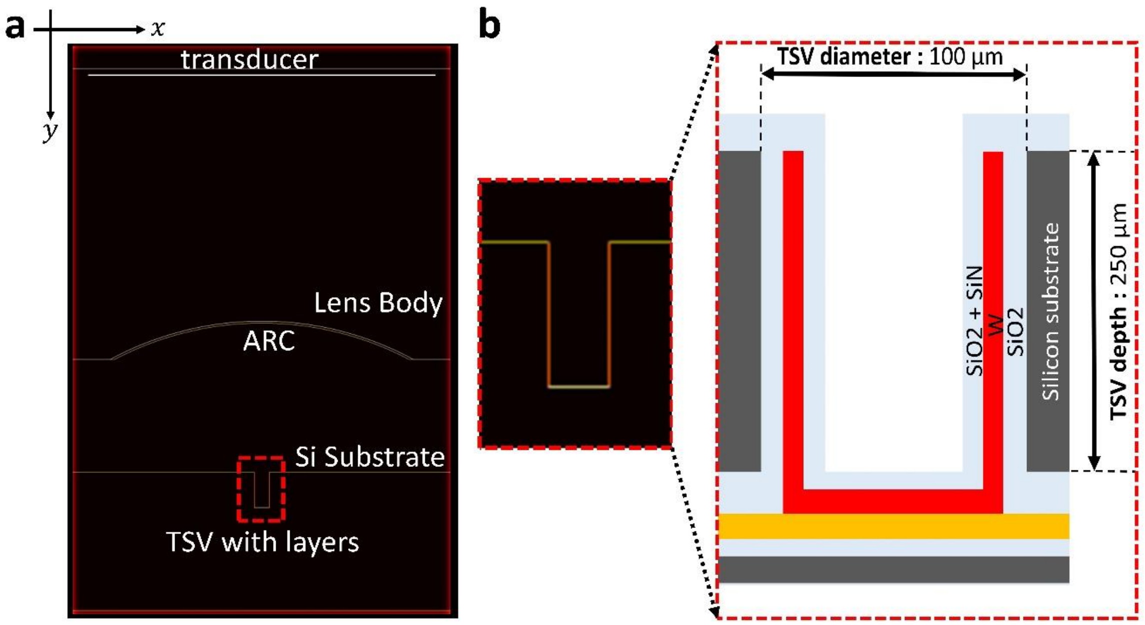

Supplementary Figure 6. Simulated SAM interferometric configuration using 2D EFIT. (a) Simulated SAM interferometry setup with transducer positioned according to experiments. A small ROI marked in red rectangular dotted box is considered around TSV to show the geometry of TSV in detail. **(b)** The geometry of simulated TSV and the corresponding schematic with different layers.

Supplementary Note 4: Investigation into the excitation of SAWs in polymer filled TSV.

The feasibility of the interferometry approach on TSVs with different technology is validated by performing 2D EFIT simulation on a polymer filled TSVs (PF - TSVs), **see Supplementary Figure 7**. A transducer with 100 MHz frequency at the optimum defocus position and acoustic lens of opening angle 60° is simulated to excite acoustic waves in the medium. The sample within the simulation is defined by the silicon substrate and a PF-TSV with a diameter of 100 µm and a depth of 250 µm. Benzocyclobutene (BCB) polymer is chosen to fill the TSV considering its advantageous dielectric, thermal, and mechanical properties, along with compatibility with semiconductor processes and strong adhesion to silicon[10]. BCB is defined in the simulation domain using its material properties such as Young's modulus (2.9 GPa), Poisson ratio (0.34) and mass density (1050 kg/m^3)[11].

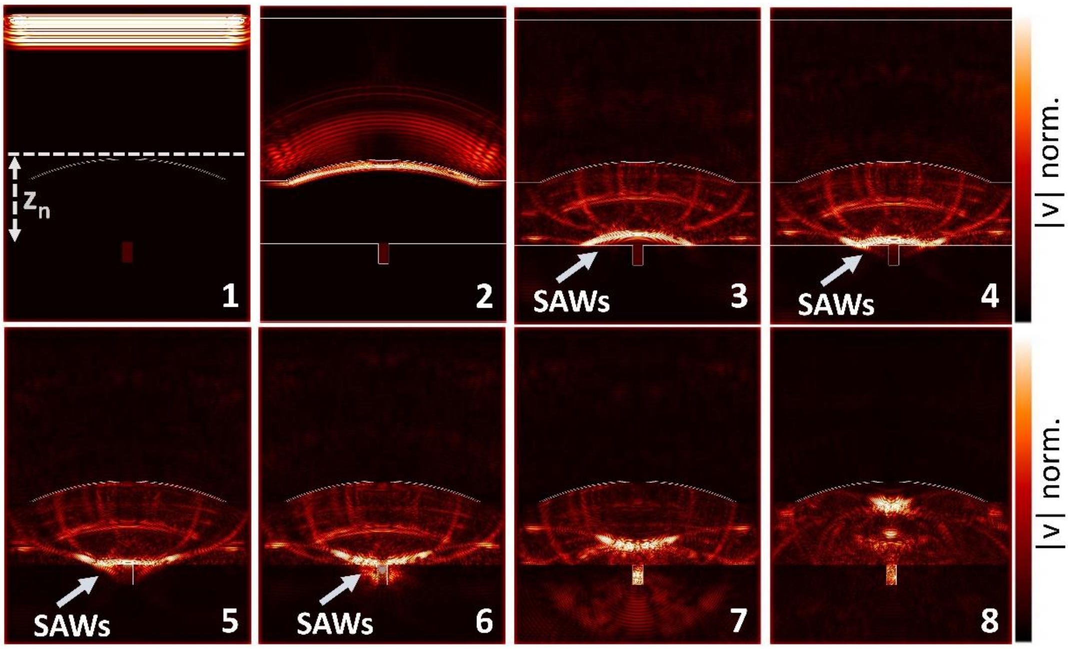

Supplementary Figure 7. Excitation of SAWs in a polymer filled TSV. The arrow marker in time step 3-6 highlights the excitation and propagation of SAWs, and time steps 7 and 8 shows the interaction of SAWs with the PF-filled TSV.

In time step 1, the plane waves excited by the 100 MHz transducer at the defocus position (Z_n) is propagating towards the ARC within the 60° opening angle of the acoustic lens. These acoustic waves further propagate towards the PF-TSV sample in time step 2. Similar to the scenario observed in non-filled TSVs, SAWs are generated near the substrate surface in time step 3. These SAWs then propagate towards the PF-TSV in time steps 4 – 5 and interact with the TSV in time steps 6-8.

This EFIT simulation confirms the excitation of SAWS and a similar behaviour as seen for the non-filled TSV. Once surface acoustic waves can be excited, an interaction with the TSV structure is possible. Therefore, we argue that, due to the interaction of the SAWs with the TSV environment, possible inhomogeneities can be detected also within the filled TSV.

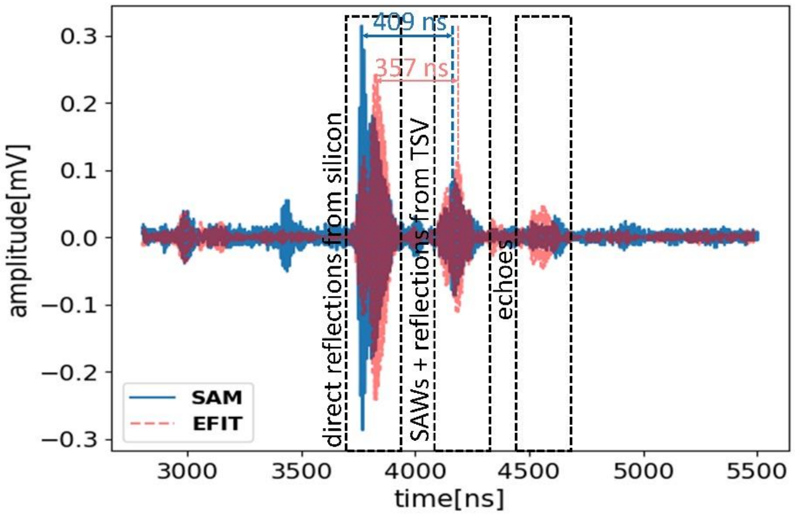

Supplementary Figure 8. Comparing Simulated A-scan signal with SAM A-scan signal at defocus position (Zn). SAM A-scan and 2D-EFIT simulated A-scan signals at defocus position (Z_n) of lens with respect to transducer.

A consistent and distinct echo pattern is evident when comparing EFIT simulated A-scan signals (depicted in blue) with SAM measured A-scan signals (shown in red color). Nevertheless, we can observe a tolerable time difference of 52 ns between the first and second reflections in the A-scan signals. This time lag may be attributed to certain approximations employed in the EFIT simulations compared to SAM experiments. These approximations encompass minor disparities in material properties, the simplifications modelling the actual TSV layers and assumptions made regarding the geometry of TSV structure (e.g., TSV scallops are not simulated).

Supplementary Note 5: 3D EFIT simulations of SAM measurement with two TSV configuration showing the dependence of transducer position with SAWs excitation and interaction.

Three-dimensional EFIT simulations were conducted to study Surface Acoustic Waves (SAWs) interaction using a dual TSV configuration. The results obtained from 3D simulations involving a dual TSV configuration, as depicted in **Supplementary Figure 9**, serve to confirm the significance of transducer placement relative to the xy axis of the TSV in inducing SAWs irrespective of the pitch length between the TSVs.

In the simulation setup, the two TSVs, labeled as 'TSV1' and 'TSV2', are deliberately positioned approximately 350 µm apart, aligning with the wafer's TSV positions. Notably, 'TSV1' is precisely centered along the central curvature of the 100 MHz, 60° AO. In a sequential timeframe spanning from step 1 to 4, we observe the progressive propagation of excited acoustic waves towards the substrate and the subsequent interaction with the substrate becomes apparent at step 5. It becomes evident that the surface acoustic waves (SAWs) generated near the substrate's surface at this juncture travel towards 'TSV2'. Intriguingly, during steps 6 to 10, these SAWs continue their trajectory, primarily interacting with 'TSV1'

and displaying zero interaction with 'TSV2'. **Supplementary Figure 9** encapsulates the simulation outcome, illuminating the link between SAW excitation and the transducer positioning. Based on these simulation findings, we posit two key points. Firstly, the interaction of SAWs is inherently contingent on the positioning of the lens in relation to the TSV. In other words, the interaction of SAWs depends on the lens positioning relative to the TSV irrespective of the pitch length between the adjacent TSV and furthermore in the excitation of SAWs. Secondly, we point out that the precise juncture at which SAWs interact with the metallized TSV wall is substantially influenced by the aperture angle of the acoustic lens.

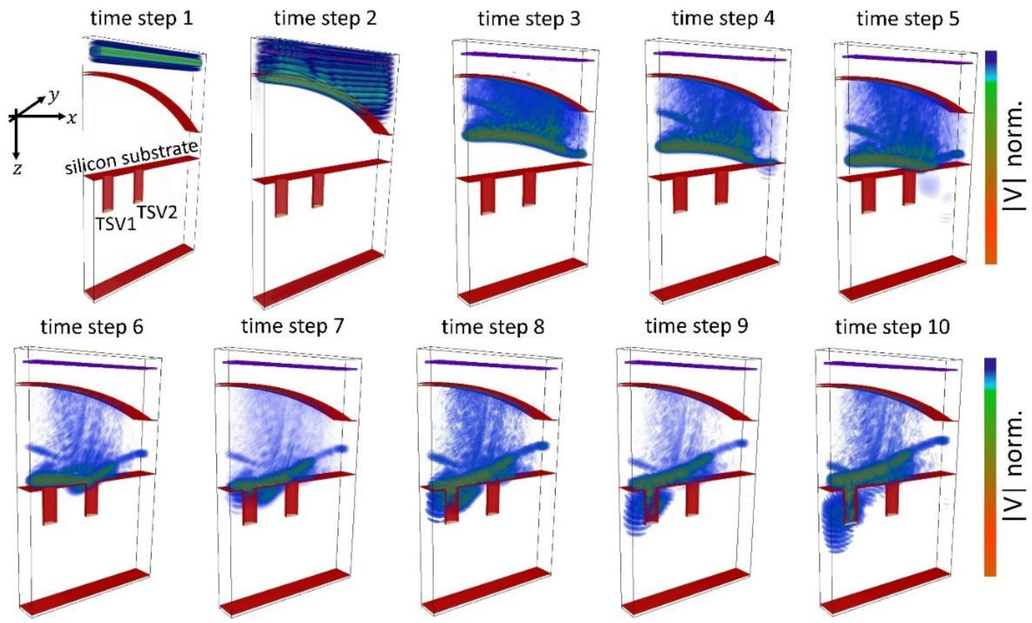

Supplementary Figure 9. The simulated acoustic lens geometry features an Anti-Reflective Coating (ARC) with an opening aperture of 60° and is affixed to a transducer operating at a central frequency of 100 MHz. The simulation demonstrates the dependence of SAWs excitation on the transducer's placement. When the transducer is precisely centred above TSV1, SAWs are excited and propagate towards TSV1, and interacting with it, see time steps 6 - 10.

SUPPLEMENTARY REFERENCES

1. Briggs, A., Briggs, G. A. D. & Kolosov, O. *Acoustic microscopy*. vol. 67 (Oxford University Press, 2010).
2. Yamanaka, K. & Enomoto, Y. Observation of surface cracks with scanning acoustic microscope. *J. Appl. Phys.* **53**, 846–850 (1982).
3. Khaled, A., Brand, S., Kögel, M., Appenroth, T. & De Wolf, I. Investigating stress measurement capabilities of GHz Scanning Acoustic Microscopy for 3D failure analysis. *Microelectron. Reliab.* **64**, 336–340 (2016).

4. Fellinger, P., Marklein, R., Langenberg, K. J. & Klaholz, S. Numerical modeling of elastic wave propagation and scattering with EFIT—elastodynamic finite integration technique. *Wave motion* **21**, 47–66 (1995).

5. Marklein, R. *The finite integration technique as a general tool to compute acoustic, electromagnetic, elastodynamic, and coupled wave fields*. (IEEE Press and John Wiley and Sons, New York, NY, USA, 2002).

6. Leckey, C. A. C., Rogge, M. D., Miller, C. A. & Hinders, M. K. Multiple-mode Lamb wave scattering simulations using 3D elastodynamic finite integration technique. *Ultrasonics* **52**, 193–207 (2012).

7. Bingham, J. & Hinders, M. 3D elastodynamic finite integration technique simulation of guided waves in extended built-up structures containing flaws. *J. Comput. Acoust.* **18**, 165–192 (2010).

8. Tschöke, K. & Gravenkamp, H. On the numerical convergence and performance of different spatial discretization techniques for transient elastodynamic wave propagation problems. *Wave Motion* **82**, 62–85 (2018).

9. Schubert, F., Peiffer, A., Köhler, B. & Sanderson, T. The elastodynamic finite integration technique for waves in cylindrical geometries. *J. Acoust. Soc. Am.* **104**, 2604–2614 (1998).

10. Duval, F. F. C., Okoro, C., Civale, Y., Soussan, P. & Beyne, E. Polymer filling of silicon trenches for 3-D through silicon vias applications. *IEEE Trans. Components, Packag. Manuf. Technol.* **1**, 825–832 (2011).

11. Jin, J., Zhao, W.-S., Wang, D.-W., Zhou, L. & Yin, W.-Y. Multiphysics characterization of polymer-filled through-silicon vias (PF-TSVs) for three-dimensional integration. *Int. J. Numer. Model. Electron. Networks, Devices Fields* **31**, e2348 (2018).
