# Supplementary material for: Fast in-line failure analysis of sub-micron-sized cracks in 3D interconnect technologies utilizing acoustic interferometry
Source: Commun Eng. 2024 Jul 19;3:100. doi: 10.1038/s44172-024-00247-8 (PMC11271500; doi:10.1038/s44172-024-00247-8)
Supplement: Supplementary file 3 — Description of Additional Supplementary Files [file 44172_2024_247_MOESM3_ESM.pdf]

Description of Additional Supplementary Files

File name: Supplementary Movie 1 (100MHz, 60 degree lens)

Description: The depicted movie shows the 3D EFIT simulation results using SAM interferometry setup. The movie commences with the generation of plane waves from a transducer positioned at a height of -140 µm from the sample surface (Z_7). These waves then propagate towards the surface of the sample. Furthermore, the movies shows the 3D simulation results of the excitation of SAWs and its interaction with the TSV. This intricate process is initiated by the use of a specialized 100 MHz transducer with a 60-degree curved acoustic lens, precisely engineered for SAWs excitation.

File name: Supplementary Movie 2 (100MHz, 80-degree lens)

Description: The movie presents the 3D EFIT simulation results, showing the generation of SAWs using a 100 MHz, 80-degree acoustic lens. The transducer is strategically positioned at same defocus height as demonstrated in 'Supplementary Movie 1 (100MHz, 60-degree lens).mp4'. i.e., at a height of -140 µm from the sample surface (Z_7). The key distinction with the 80-degree acoustic lens is its closer proximity to the sample surface due to its more pronounced curvature. The movie offers a comprehensive view of the entire interferometry process, including the plane wave excitation by the transducer, the propagation of waves towards the sample surface, and various steps from the excitation of SAWs and their interaction with the TSV height.
